# Systemic inflammation and risk of multiple sclerosis – A presymptomatic case-control study

**DOI:** 10.1177/20552173221139768

**Published:** 2022-11-22

**Authors:** Viktor Grut, Martin Biström, Jonatan Salzer, Pernilla Stridh, Anna Lindam, Lucia Alonso-Magdalena, Oluf Andersen, Daniel Jons, Martin Gunnarsson, Magnus Vrethem, Johan Hultdin, Peter Sundström

**Affiliations:** Department of Clinical Science, Neurosciences, Umeå University, Umeå, Sweden; Department of Clinical Neuroscience, 27106 Karolinska Institutet, Stockholm, Sweden; Center for Molecular Medicine, 59562 Karolinska University Hospital, Stockholm, Sweden; Department of Public Health and Clinical Medicine, Unit of Research, Education and Development Östersund Hospital, Umeå University, Umeå, Sweden; Department of Neurology, Skåne University Hospital and Department of Clinical Sciences, Neurology, 5193 Lund University, Lund, Sweden; Department of Clinical Neuroscience, Institute of Neuroscience and Physiology, 70712 Sahlgrenska Academy, University of Gothenburg, Gothenburg, Sweden; Department of Neurology, Faculty of Medicine and Health, 6233 Örebro University, Örebro, Sweden; Department of Neurology and Department of Biomedical and Clinical Sciences (BKV), 4566 Linköping University, Linköping, Sweden; Department of Medical Biosciences, Clinical Chemistry, Umeå University, Umeå, Sweden; Department of Clinical Science, Neurosciences, Umeå University, Umeå, Sweden

**Keywords:** Case-control studies, C-reactive protein, systemic inflammation, multiple sclerosis

## Abstract

**Background:**

C-reactive protein (CRP) is a marker of systemic inflammation. Increased levels of CRP in young persons have been suggested to decrease the risk of multiple sclerosis (MS).

**Objectives:**

To assess CRP as a risk factor for MS.

**Methods:**

Levels of CRP were measured with a high-sensitive immunoassay in biobank samples from 837 individuals who later developed MS and 984 matched controls. The risk of developing MS was analysed by conditional logistic regression on *z*-scored CRP values.

**Results:**

Levels of CRP were not associated with MS risk.

**Conclusions:**

We found no association between CRP levels and risk of MS development.

## Introduction

Multiple sclerosis (MS) is an autoimmune disease affecting the central nervous system.

According to the prevailing hypothesis, MS is caused by a complex interplay of environmental risk factors and genetic predispositions.^1^ Certain viral infections are now broadly accepted risk factors for MS, but the effect of exposure appears to be time-dependent.^2^ The hygiene hypothesis suggests that childhood exposure to infections could contribute to the development of the immune system and protect against autoimmune diseases such as MS.^2^ We previously reported that increased levels of C-reactive protein (CRP) among young individuals were associated with a lower risk of developing MS, which may support the hygiene hypothesis.^3^ However, the sample size was limited, and the results have not been replicated in a larger prospective study.

The aim of the present study was to assess CRP as a risk factor for MS in a large study on presymptomatically collected samples.

## Methods

Plasma or serum from individuals later developing relapsing-remitting MS were identified and retrieved by cross-linkage of Swedish MS registries and six Swedish microbiological biobanks, with samples collected in a clinical setting, previously described in detail.^4^ Samples were donated before MS symptoms and before the age of 40. For each case, one control without MS was randomly selected, matched for biobank, sex, date of sampling, and date of birth (in order of priority). We retrieved samples from 670 cases and 670 matched controls. Five individuals had insufficient sample volumes, and the corresponding case-control sets were excluded, leaving 665 sets for analysis constituting the primary cohort. Demographic data is available elsewhere.^5^

To increase the statistical power, we merged the datasets from the primary cohort and the previous study.^3^ In this combined cohort, duplicate cases (*n*  =  20) and possible duplicate controls (*n*  =  75, deduced from birth date) from the previous study were excluded, leaving 837 cases and 984 controls for analysis.

### Laboratory procedures

In the primary cohort, the concentration of CRP was analysed by multiplex immunoassay (V-PLEX Vascular Injury Panel 2 Human Kit, Mesoscale), and in the previous study with an ELISA (Immundiagnostik AG, Bensheim, Germany). Both methods were high-sensitive. The samples from matched cases and controls were analysed consecutively but randomly. Case-control status was blinded for the technicians.

### Statistical methods

Levels of CRP were compared with Mann-Whitney *U-*test and Wilcoxon signed-rank test. The association of CRP levels and MS risk was analysed by calculating odds ratios (ORs) and 95% confidence intervals (CIs) with conditional logistic regression.

Elevated CRP was defined as ≥10 mg/L, as in the previous study.^3^ We also analysed CRP continuously using *z*-score of log_10_-transformed CRP levels. Individuals with CRP levels below the quantification range (*n*  =  80) were assigned a value of half the lower level of detection for the assay. Since the CRP levels differed significantly between men and women (*p*  =  0.03) and between biobanks (*p*  =  0.01), *z*-scores were calculated separately for each biobank and sex. *Z-*scores were also calculated separately for the samples from the previous study, which used a different CRP assay. These values were then used in a pooled analysis. Analyses were stratified based on age at blood sampling:  < 20, 20–29 and >30 years of age. Subgroup analyses were performed in samples drawn for (1) screening (*n*  =  572 cases and 719 controls) and (2) diagnosis of acute disease (*n*  =  265 cases and 265 controls). These analyses were also stratified by sampling age, using the cut-off of 26.4 years, as in the previous study.

Statistical analyses were performed with IBM SPSS version 27.

### Ethical considerations

The regional ethical review board in Umeå approved this study (2011-198-31M with amendments). No written informed consent was required.

## Results

**Figure 1.**
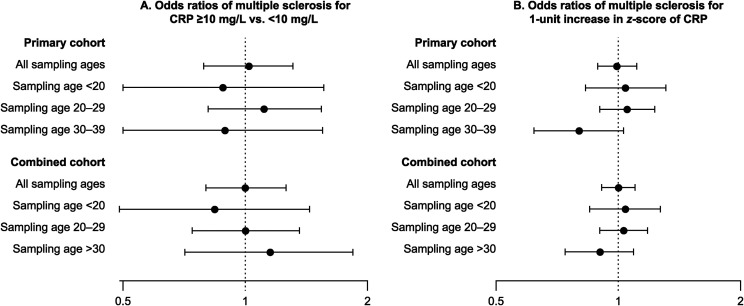
Odds ratios with 95% confidence intervals for multiple sclerosis by age strata and cohort.

The median CRP levels did not differ significantly between cases and controls in any age strata in the primary or the combined cohort (Table 1). We did not observe significant associations between elevated CRP or CRP *z-*score and MS risk in any of these groups (Figure 1). Neither did the subgroup analyses yield significant results (data not shown).

Primary cohort: Presymptomatic biobank samples of 665 cases and 665 matched controls from six Swedish microbiological biobanks; Combined cohort: Presymptomatic biobank samples of 837 cases and 984 controls from current and previous studies. CRP *z*-score: *Z*-score of log_10_-transformed CRP, calculated separately for each biobank and sex.

**Table 1.** Levels of CRP among cases and controls by age strata and cohorts.

By sampling age:	*N* (%)	CRP (mg/L)		*p* ^a^
		Cases	Controls	
All ages	1330 (100%)	3.4 (1.1–10.7)	3.5 (1.1–10.1)	0.89
Age <20	282 (21.2%)	3.9 (1.1–10.7)	3.2 (0.9–12.5)	0.98
Age 20–29	750 (56.4%)	3.4 (1.1–10.7)	3.4 (1.1–9.7)	0.92
Age 30–39	298 (22.4%)	3.1 (1.0–10.5)	4.3 (1.2–11.1)	0.73
By biobank:	*N* (%)	Cases	Controls	*p* ^a^
Umeå	204 (15.3%)	1.8 (0.6–10.8)	2.5 (0.9–7.1)	0.65
PHAS	276 (20.8%)	3.5 (1.2–9.7)	3.6 (1.0–13.1)	0.49
Örebro	58 (4.4%)	4.3 (1.8–8.8)	5.3 (2.6–12.9)	0.63
Gothenburg	94 (7.1%)	3.9 (0.8–7.5)	3.3 (1.1–13.2)	0.17
Skåne	620 (46.6%)	3.6 (1.3–11.3)	3.3 (0.9–9.1)	0.39
Linköping	78 (5.9%)	3.5 (1.2–11.4)	5.8 (2.4–15.7)	0.45
Combined cohort:	Cases/Controls (*n*)	Cases	Controls	*p* ^b^
All ages	837/984	3.0 (0.8–8.7)	2.7 (0.8–8.4)	0.20
Age <20	164/179	3.6 (0.8–9.7)	2.5 (0.6–10.1)	0.34
Age 20–29	466/546	3.0 (1.0–8.7)	2.9 (0.9–8.4)	0.52
Age >30	207/259	2.2 (0.6–8.5)	2.4 (0.6–7.5)	0.55

Values for CRP represent median (25^th^–75^th^ percentile). PHAS: Public Health Agency of Sweden.

^a^ Calculated with Wilcoxon signed-rank test (1:1 matching).

^b^ Calculated with Mann-Whitney *U*-test (1:*N* matching).

## Discussion

In this study, we analysed CRP levels as a potential risk factor for developing MS. The purpose was to replicate our previous study, where high CRP levels among young individuals were associated with a lower risk of MS. We used a similar methodological approach but increased the sample size more than four-fold using the combined datasets from the previous and the present study. However, no significant associations between increased CRP levels and MS were observed. The previous finding was observed in a subgroup and not adjusted for multiple comparisons; random error is thus a possible explanation.

Although large, the present study has limitations. As no clinical data were available, confounders such as obesity, smoking, acute infections or the use of medications could not be accounted for. The samples derive from six biobanks, using differing storage procedures. These factors are likely explanations for the different CRP levels between the biobanks. However, the control matching within each biobank and the use of biobank-specific *z*-scores reduce the effect of these factors. The biobank samples also have different inclusion criteria: many samples were collected to diagnose acute disease, which could attenuate possible differences between cases and controls. In contrast, most of the samples in the previous study were collected from pregnant women at a maternity clinic, and the remaining from population-based health programs. This difference in sample composition might affect the results in the present study. To match the sample composition of the previous study, we made subgroup analyses restricted to samples drawn for screening. These analyses indicated no association between CRP and MS risk: OR of MS for CRP *z*-score: 1.14 (95% CI 0.97–1.34).

In conclusion, this study could not replicate the previous finding that increased CRP levels at a young age could be associated with a lower MS risk. While systemic inflammation remains of interest in the aetiopathogenesis of MS, an association with MS risk was not found using CRP as a marker.
